# Diversity of lactic acid bacteria of the bioethanol process

**DOI:** 10.1186/1471-2180-10-298

**Published:** 2010-11-23

**Authors:** Brigida TL Lucena, Billy M dos Santos, João LS Moreira, Ana Paula B Moreira, Alvaro C Nunes, Vasco Azevedo, Anderson Miyoshi, Fabiano L Thompson, Marcos Antonio de Morais

**Affiliations:** 1 Institute of Biology, Federal University of Rio de Janeiro (UFRJ), Rio de Janeiro, Brazil; 2 Department of Genetics, Federal University of Pernambuco (UFPE), Recife, Brazil; 3 Department of General Biology, Federal University of Minas Gerais (UFMG), Belo Horizonte, Brazil

## Abstract

**Background:**

Bacteria may compete with yeast for nutrients during bioethanol production process, potentially causing economic losses. This is the first study aiming at the quantification and identification of Lactic Acid Bacteria (LAB) present in the bioethanol industrial processes in different distilleries of Brazil.

**Results:**

A total of 489 LAB isolates were obtained from four distilleries in 2007 and 2008. The abundance of LAB in the fermentation tanks varied between 6.0 × 10^5 ^and 8.9 × 10^8 ^CFUs/mL. Crude sugar cane juice contained 7.4 × 10^7 ^to 6.0 × 10^8 ^LAB CFUs. Most of the LAB isolates belonged to the genus *Lactobacillus *according to rRNA operon enzyme restriction profiles. A variety of *Lactobacillus *species occurred throughout the bioethanol process, but the most frequently found species towards the end of the harvest season were *L. fermentum *and *L. vini*. The different rep-PCR patterns indicate the co-occurrence of distinct populations of the species *L. fermentum *and *L. vini*, suggesting a great intraspecific diversity. Representative isolates of both species had the ability to grow in medium containing up to 10% ethanol, suggesting selection of ethanol tolerant bacteria throughout the process.

**Conclusions:**

This study served as a first survey of the LAB diversity in the bioethanol process in Brazil. The abundance and diversity of LAB suggest that they have a significant impact in the bioethanol process.

## Background

Bioethanol is a profitable commodity as renewable energy source. Brazil is the second largest bioethanol producer of the planet, with a production of 16 billion liters per year. The 360 active Brazilian distilleries use sugarcane juice and/or sugar molasses (12-16° Brix in the wort) as substrates for fermentation by *Sacharomyces cerevisiae *[1-3]. Several factors may influence the yield of the process, including (i) management, (ii) low performance of the yeast, (iii) quality of the sugarcane juice and molasses, and (iv) microbial contamination. The bioethanol process should be developed in septic conditions during all the production period. One of the most common strategies to control microbial contamination is the cleaning of the fermentation tanks and disinfection of the yeasts. Yeast cells are re-used during the six months of the harvest season [4]. In the end of each fermentation cycle, which takes between 8 and 10 hr, yeast cells are collected and transferred to pre-fermenter tanks where they are washed in aqueous sulfuric acid solution in order to reduce bacterial contamination. This type of treatment may cause serious metabolic stress in the yeast cells, decreasing their viability [5]. Another alternative to control microbial contamination is the pre-treatment of the fermentation substrate (sugar cane juice and molasses) by pasteurization. It can reduce bacterial contamination to lower levels (ca. 10^3 ^cells/ml), but the high costs for cooling the substrate is not economically viable. Industrial antibiotics are also frequently used by many distilleries in the pre-fermentation stage, in spite of possible environmental impacts they may cause [4].

Bacterial contamination appears to reduce the process productivity, by reducing yeast growth, viability, and fermentation capacity [6,7]. Lactic Acid Bacteria (LAB) are very abundant in the bioethanol process possibly because of their tolerance to ethanol, low pH and high temperature [8]. Lactic and acetic acids produced by LAB may interfere in the yeast metabolism [8]. Proliferation of LAB in the fermentation tanks is often unpredictable, leading to shut down of the refinery for cleaning and desinfection. The proliferation of LAB has indeed a negative effect in the process and may cause serious economic losses. Therefore, it is crucial to have a better understanding of the abundance and diversity of LAB throughout the bioethanol process in order to design more efficient production processes. To our knowledge, this is the first study in Northeast Brazilian distilleries aiming at the characterization of the bioethanol process microbiota. The aim of the present study was to analyze the abundance and diversity of LAB in the bioethanol process. Four representative distilleries (Japungu, Miriri, Giasa and Trapiche) in Northeast Brazil were monitored between 2007 and 2008.

## Results

**Figure 1:**
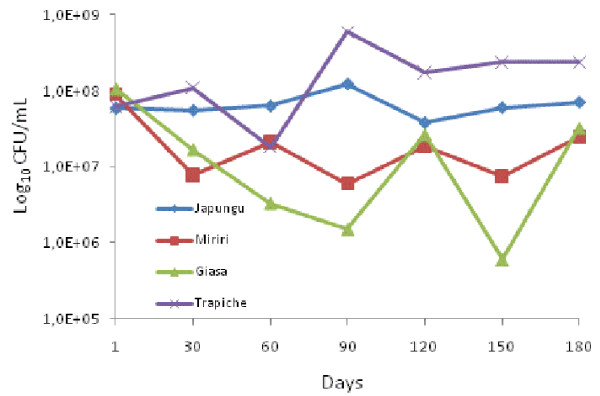
**Mean abundance of LAB CFUs in the four refineries during the bioethanol process each 30 days**. Log_10 _CFU counts.

**Figure 2:**
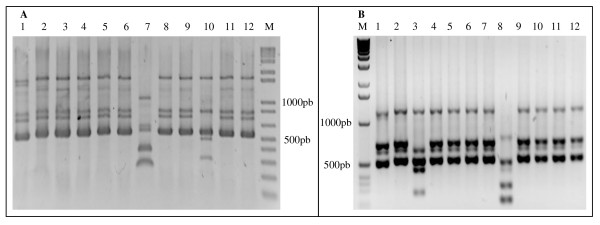
**Restriction profile of the intergenic 16S-23S region of the *Lactobacillus vini *(A) and *Lactobacillus fermentum *(B) with the enzymes *Sph*I (lane 1), *Nco*I (lane 2), *Nhe*I (lane 3), *Ssp*I (lane 4), *Sfu*I (lane 5), *Eco*RV (lane 6), *Dra*I (lane 7), *Vsp*I (lane 8), *Hin*cII (lane 9), *Eco*RI (lane 10), *Hin*dIII (lane 11) and *Avr*II (lane 12)**. M, 1 Kb molecular marker.

The total mean number of CFUs in Japungu, Miriri, Giasa and Trapiche varied between 3.7 × 10^7 ^and 1.2 × 10^8^, 7.5 × 10^6 ^and 8.9 × 10^7^, 6.0 × 10^5 ^and 8.9 × 10^8^, and 1.8 × 10^7 ^and 5.9 × 10^8^, respectively (Figure 1). Crude sugar cane juice contained 7.4 × 10^7 ^to 6.0 × 10^8 ^LAB CFUs. Juice cane LAB isolates were not identified in this study. Ethanol content in the process varied between 5.9 and 7.9%. A total of 489 putative LAB isolates were obtained from the fermentation tanks of four distilleries (additional file 1). The screening of the 489 presumptive LAB isolates by means of restriction enzyme analysis of rRNA operon allowed the rapid presumptive identification of the species found in the bioethanol process. The detailed reference restriction pattern of each species (additional file 2) and examples of *L. vini *and *L. fermentum *patterns are presented (Figure 2). The typical patterns contained three diagnostic bands (between 500 and 1000 bp). Most of the isolates were identified in the genus *Lactobacillus *in the fermentation process in the four distilleries by means of their Amplified Ribosomal DNA Restriction Analysis (ARDRA) pattern. Only 13 isolates remained as unidentified LAB.

**Figure 3:**
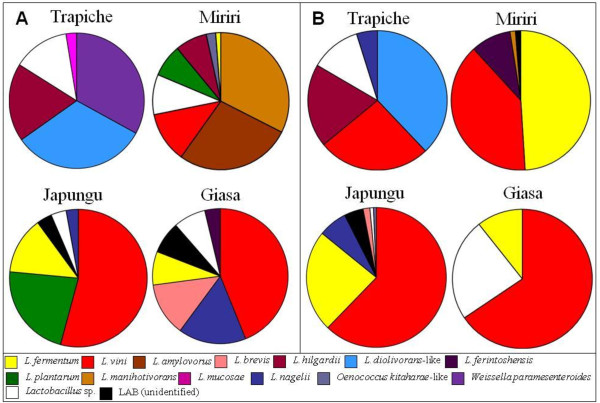
**Percentage of isolates of each LAB species found in the beginning (A) and towards the end of the process (B)**. Panel A was based on the samples of days 1 and 30 of the process. Panel B was based on all remainder samples (at 60, 90, 120, 150 and 180 days of process). The graphs show the percentage of species in Trapiche (N = 100), Miriri (N = 111), Japungu (N = 180), and Giasa (N = 98).

**Figure 4:**
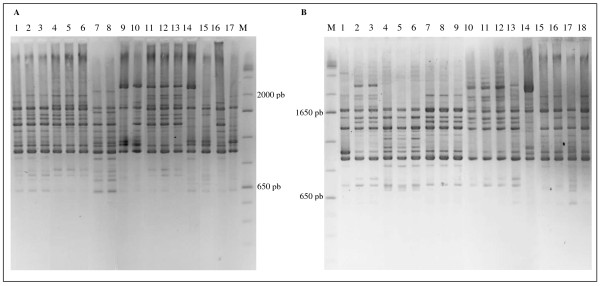
**Rep-PCR patterns of 35 *Lactobacillus fermentum *isolates obtained from Miriri (A) Japungu and Giasa (B)**. M7.3.9 (Lane A1), M7.3.10 (Lane A2), M7.3.11 (Lane A3), M7.3.14 (Lane A4), M7.3.15 (Lane A5), M7.3.16 (Lane A6), M7.3.7 (Lane A7), M7.3.8 (Lane A8), M7.4.6 (Lane A9), M7.4.8 (Lane A10), M7.3.17 (Lane A11), M7.3.19 (Lane A12), M7.3.20 (Lane A13), M7.4.1 (Lane A14), M7.4.3 (Lane A15), M7.3.12 (Lane A16), M7.4.9 (Lane A17), JP7.2.9 (Lane B1), JP7.5.1 (Lane B2), JP7.5.9 (Lane B3), JP7.6.7 (Lane B4), JP7.6.8 (Lane B5), JP7.6.9 (Lane B6), JP7.6.10 (Lane B7), JP7.6.11 (Lane B8), JP7.6. 12 (Lane B9), JP7.2.10 (Lane B10), JP7.2.11 (Lane B11), JP7.3.12 (Lane B12), JP7.3.20 (Lane B13), JP7.4.19 (Lane B14), G7.4.10 (Lane B15), G7.4.11 (Lane B16), G7.6.13 (Lane B17), G7.6.18 (Lane B18). M, 1 Kb molecular weight.

**Figure 5:**
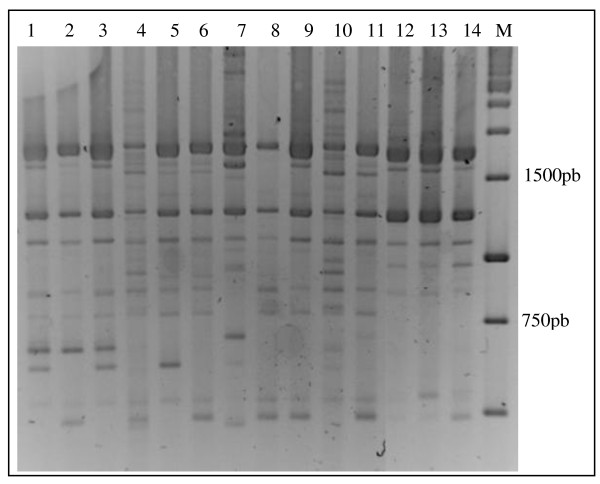
**Rep-PCR patterns of 14 *Lactobacillus vini *obtained from Miriri, Trapiche, Japungu, and Giasa**. JP7.3.2(Lane 1), JP7.4.3 (Lane 2), JP7.3.7* (Lane 3), JP7.5.18 (Lane 4), M7.3.2 (Lane 5), M.7.3.3 (Lane 6), M7.6.11(Lane 7), M7.7.5 (Lane 8), G.7.2.19 (Lane 9), G7.4.2 (Lane 10), G7.3.2 (Lane 11), TR7.5.7* (Lane 12), TR7.5.13* (Lane 13) and TR7.5.15* (Lane 14). M, 1 Kb molecular weight. *, isolates also identified by *pheS *sequences.

There was a higher number of LAB species in the first 30 days of the fermentation process (Figure 3A). *Lactobacillus plantarum *was frequently found in the beginning of the fermentation process at Miriri and Japungu distilleries. *L. manihotivorans *was found in the beginning of the fermentation process at Miriri, whereas *Weissella paramesenteroides *was found at Trapiche. Overall, there was a predominance of *L. fermentum *and *L. vini *after 60 days of fermentation. The two species, *L. fermentum *and *L. vini*, corresponded to the majority of the isolates obtained in this study (Figure 3B). There was a tendency of reduction of the LAB species numbers towards the end of the process, suggesting the occurrence of antibiotic resistance and/or the occurrence of persistent endemic infections. The harsh conditions of the process (antibiotics, high temperature, low pH, and high ethanol concentration) possibly have a selective pressure over the microbiota, leading to a selection of certain resistant LAB types. *L. ferintoshensis*, *L. diolivorans*-like, *L. nagelii*, unidentified LAB, and *Oenococcus kitaharae*-like were also found at the end of the fermentation process. Trapiche distillery showed the most distinct LAB composition possibly due to the sole use of molasses. The presumptive identification based on restriction enzyme analysis of rRNA was confirmed for several *L. vini *and *L. fermentum *isolates using *pheS *and 16S rRNA gene sequences (data in attached; GenBank under the accession nos. HQ009762-HQ009795; additional file 3). For instance, the isolates JP7.3.7, TR7.5.7, TR7.5.13, TR7.5.15 had > 99% *pheS *sequence similarity towards the *L. vini*. *Oenococcus kitaharae*-like isolates and *Lactobacillus *sp. isolates were also tentatively identified by gene sequences, confirming their status of unknown species. Rep-PCR analysis using GTG5 primers was performed in order to evaluate the intra-specific diversity in *L. fermentum *and *L. vini*. Representative isolates of the species *L. fermentum *from the four distilleries obtained in the same and in different sampling periods had distinct fingerprint patterns, indicating a high genomic diversity of co-occurring populations (Figure 4). Likewise, representative *L. vini *isolates had different patterns (Figure 5). The high genomic diversity observed in *L. fermentum *and *L. vini *during the fermentation process in the four distilleries suggested the co-occurrence of several populations possibly through the introduction of new types via substrate carriage during the process. These populations are possibly adapted to tolerate ethanol. Representative *L. fermentum *and *L. vini *isolates obtained in this study grew in broth containing up to 10% ethanol, reaching 10^6 ^cells/mL in48 hours of experiment in the laboratory. In the control treatments, cells grown in broth without ethanol addition reached the same densities in less than 24 hours.

## Discussion

This study demonstrates that LAB is commonly found in the bioethanol process in Brazilian distilleries. Fermentation substrates (sugar cane and molasses) appear to be important sources of contamination. The bacterial abundance in substrates depends on several factors, including the origin of the cane, the time from harvesting to smashing and the rate of rain in the period [1,9]. The dominance of *L. vini *and *L. fermentum *after 30 days of the fermentation process indicates that these two species are highly adapted to the bioethanol process. *L. fermentum *may induce flocculation of yeast cells [10]. The species *L. vini *was recently classified based on a group of isolates originated from fermented grape musts [11]. It is related to *L. nagelii *and *L. satsumensis*. *L. vini *is physiologically versatile, having a facultative anaerobic homofermentative metabolism, the ability to ferment hexoses and pentoses (ribose and arabinose) to lactic acid, and growth between 25°C and 45°C. The diversity of LAB has been characterized in other types of fermentation processes. In the United States, the fermentation process uses corn starch or fiber hydrolysates as substrate for fermentation. In this process, *L. acidophilus*, *L. agilis*, *L. amylovorus*, *L. brevis*, *L. casei, L. hilgardii*, *L. fermentum, L. plantarum *and *W. paramesenteroides *are commonly found [6,7]. The bacterial diversity was also analyzed in ethanol fermentation processes in Vietnam [12]. *L. brevis*, *L. plantarum*, *Pediococcus pentosaceus, Weissella confusa *and *W. paramesenteroides *were the most frequently found LAB. Moreover, acetic acid bacteria (*Acetobacter orientalis *and *A. pasteurianus*), amylase-producing bacteria (*Bacillus subtilis, B. circulans, B. amyloliquefaciens *and *B. sporothermodurans*) and some plant pathogen bacteria (*Burkholderia ubonensis, Ralstonia solanacearum *and *Pelomonas puraquae*) were also reported. The species *Lactobacillus vini *was observed in association with the growth of the yeast *Dekkera bruxellensis *in a Swedish bioethanol refinery [13]. This process passed by a period of decrease in fermentation before stabilization. The present study also found a high abundance of *Dekkera bruxellensis *(10^7 ^CFUs/mL), possibly indicating an association between this yeast and LAB.

Effects of LAB on *Sacharomyces cerevisiae *viability were reported by the inoculation of *L. fermentum *and *L. delbrueckii *in wheat mash batch fermentation [14]. *Lactobacillus paracasei *was reported to affect yeast viability when lactic acid concentration in the process exceeded 8 g/L [15]. This effect is more pronounced when in combination with acetic acid [16]. Induction of yeast flocculation has been associated with some *L. fermentum *strains in synergy with the presence of calcium, which leads to loss of yeast viability [17]. Decrease of yeast cell viability was also induced by inactivated cells of *L. fermentum*, suggesting that bacterial metabolites can interfere in the yeast population [18]. Strains of *L. plantarum, L. fructivorans, L. fructosus *and *L. buchneri *were also able to induce yeast flocculation depending on the cell density [19,20]. Experiments performed at laboratory scale simulating the contamination with *L. fermentum *showed that viability of the yeast cells, sugar consumption and ethanol yield were severely affected when acetic acid was higher than 4.8 g/L [10]. In the present work observations such as the microbiota alterations throughout the process, the presence of distinct populations of *L. vini *and L. *fermentum*, and the co-ocurrence of high numbers of *D. bruxellensis *and *L. vini *indicate a complex microbial ecology in the bioethanol process. The abundance and diversity of LAB observed in the four distilleries analyzed in this study also suggest that the bioethanol process can be improved through the use of (i) higher quality raw material (sugar cane), mainly fresh plants with low loads of soil and microbes, (ii) washing of sugar cane plants with clean water, (iii) higher quality substrates (cane juice and molasses) that would contain a lower load of LAB, and (iv) strain-specific designed antimicrobial treatments targeting the most frequently found LAB. Often harvesting of sugar cane plants is uncoupled of the subsequent steps of the process (e.g. juice production), resulting in the partial rooting of the plants and microbial growth. The high CFU counts obtained in this study suggest that contamination is usual in the bioethanol process. The genomic variability observed in rep-PCR patterns indicates the re-inoculation of different types of *L. fermentum *and *L. vini *throughout the process possibly due to the management practices.

Because industrial data of the four distilleries examined in this study suggested that lactic acid concentration in the fermentation process was high, and considering that LAB was reported as a major component of the microbiota of the bioethanol process in other studies [6,7], we used an elective general medium that allows growth of LAB to isolate the highest number of this type of bacteria. It is important to notice that MRS recovered different types of LAB. This medium was not selective for a given type of LAB, suggesting that it recovered a wide variety of circulating LAB types. Although, we cannot rule out the possibility that some LAB were overlooked in this study, but in any case we consider that this study gives an initial contribution to the field.

## Conclusions

This is the first study aiming at a broad survey of LAB diversity in the bioethanol process in Brazil. The results herein presented clearly illustrate that LAB are an important component of the bioethanol process. Improved management practices may increase the yields of the bioethanol process. This study opens up new avenues of research aiming at the control and technological use of LAB. Due to their ability to grow in harsh environmental conditions, these bacteria may offer new genes and pathways for technological applications. In addition, detailed taxonomic work underway will describe the new species found in the bioethanol process.

## Methods

### Strains, culture conditions and cell maintenance

The industrial samples analyzed herein were collected monthly from the fermentation tanks throughout the harvest period, beginning with the first day of fermentation up to the end of the process (180 days), in four distilleries in the harvesting season 2007-2008. Trapiche (Sirinhaém-PE, Brazil) used molasses, whereas Giasa (Pedras de Fogo-PB, Brazil), Miriri and Japungu (Santa Rita-PB, Brazil) used sugar cane juice. The four distilleries perform yeast cleanup by means of sulfuric aqueous solution in order to reduce bacterial contamination. Antibiotics (penicillin and ionophore monensin) are also commonly used in order to reduce bacterial contamination in the four distilleries. Data on ethanol production was obtained directly from the producers. Samples were collected in sterile plastic bags, transported on ice and processed in the same day by diluting in sterile saline to 3×10^-4^, and 0.1 mL of this dilution was plated onto MRS medium [21] containing cycloheximide at 0.1% to inhibit yeast growth. Plates were incubated at 37°C in anaerobic jars for 4 days. Twenty representative bacterial colony morphotypes were selected for further taxonomic identification. Isolates are maintained in glycerol 30% at -80°C. In total 7 samples (days 1, 30, 60, 90, 120, 150, and 180) were used to estimate bacterial CFU numbers in the four distilleries. Each sample was analyzed in duplicate. Ethanol tolerance test was performed with representative LAB isolates grown in MRS broth supplemented with Ethanol (100 g/L) at 37°C and pH 6.5. Cell growth was estimated by means of optical density measurement at 600 nm using a Biophotometer (Eppendorf). Diluted samples (0.1 mL) were also plated onto Wallerstein laboratory nutrient agar (WLN) medium containing 0.1% bromocresol green for the determinations of yeast abundance and presumptive identification [22].

### ARDRA fingerprinting

The fragment of the 16S-23S spacer was amplified with the primers 16-1A (5'-GAATCGCTAGTAATCG-3') that anneals to nucleotides 1361 to 1380 of 16S rRNA gene (using *L. casei *genome location) and 23-1B (5'-GGGTTCCCCCATTCGGA-3') that anneals to nucleotides 123 to 113 of 23S rRNA gene (using *L. casei *genome location) [23]. The amplification reaction contained 0.5 μM of each primer, 0.2 mM dNTP mix, 1.5 mM MgCl_2 _and 5 U Taq DNA polymerase (Invitrogen) in 50 μL final volume. The PCR amplification used a standard thermal program (two minutes at 94°C, followed by 35 cycles of 94°C for 30 seconds, 55°C for one minute and 72°C for one minute, with a final extension step at 72°C for 10 minutes). ARDRA analysis was performed using the 12 restriction enzymes *Sph*I, *Nco*I, *Nhe*I, *Ssp*I, *Sfu*I, *Eco*RV, *Dra*I, *Vsp*I, *Hinc*II, *Eco*RI, *Hind*III and *Avr*II as described previously [23]. The restriction profiles of the isolates obtained from the bioethanol process were compared to the ARDRA database reported by Moreira *et al*. [24]. The ARDRA profiles of the isolates were compared with the ARDRA database. An isolate having an ARDRA profile matching an ARDRA profile of known LAB species was identified into this species.

### pheS and 16S rRNA sequencing

The 16S rRNA was amplified by PCR using the primers 27F (5'-AGAGTTTGATCCTGGCTCAG-3') and 1492R (5'-GGTTACCTTGTTACGACTT-3') [25], while the *pheS *was amplified with the primers 21-F (5'-CAYCCNGCHCGYGAYATGC-3') and 22-R (5'-CCWARVCCRAARGCAAARCC-3') or 23-R (5'-GGRTGRACCATVCCNGCHCC-3') [26]. The reactions contained 0.5 μM each primer, 0.2 mM dNTP mix, 1.5 mM MgCl_2 _and 1 U Taq DNA polymerase (Invitrogen) in a final volume of 50 μL. Amplification and sequencing was performed as described previously [27]. Gene sequences were analyzed using the software BioEdit v7.0. *pheS *and 16S rRNA nucleotide sequences are deposited in the GenBank under the accession nos. HQ009762-HQ009795.

### REP-PCR fingerprinting

DNA fingerprinting analysis was performed using (GTG)_5 _primer as described previously [27,28]. Amplification reactions contained 0.2 pmol of the (GTG)_5 _primer, 0.2 mM dNTP mix, 3 mM MgCl_2_, 0.025 μg/μL BSA and 1 U Taq DNA polymerase (Invitrogen). The PCR thermal program (Seven minutes at 95°C, followed by 30 cycles of 95°C for one minute, 40°C for one minute and 65°C for eight minutes, and a final extension at 65°C for 16 minutes) was used as described previously [27,28]. PCR products were checked on a 1.5% agarose gel at 5 V/cm for four hours in 0.5 × TBE buffer, stained in ethidium bromide. Gel images were recorded using a PhotoCapture™ system. Similarity between patterns was determined by visual inspection.

## Authors' contributions

BTLL and BMS performed LAB isolation, rRNA restriction profiling analysis and rep-PCR; JLSM, ACN and VA participated in the rRNA restriction profiling analysis; BTLL and APBM performed ethanol tolerance tests and, 16S sequencing pheS sequencing; MAMJ and FLT funded the project, analyzed the data and wrote the manuscript. All authors read and approved the final manuscript.

## Supplementary Material

Additional file 1 **Table 1 Strain list**. Strain list with place, date, and source of isolation. Click here for file

Additional file 2 **Table 2 Restriction patterns of 16S-23S intergenic spacer of LAB from bioethanol fermentation process**. Patterns of restriction of 16S-23S intergenic spacer of LAB with 12 enzymes. Click here for file

Additional file 3 **Gene sequences**. 16S rRNA and pheS gene sequences of several representative LAB Click here for file
