# Supplementary material for: Diversity of lactic acid bacteria of the bioethanol process
Source: BMC Microbiol. 2010 Nov 23;10:298. doi: 10.1186/1471-2180-10-298 (PMC2999616; doi:10.1186/1471-2180-10-298)
Supplement: Additional file 2 — Table 2 Restriction patterns of 16S-23S intergenic spacer of LAB from bioethanol fermentation process. Patterns of restriction of 16S-23S intergenic spacer of LAB with 12 enzymes. [file 1471-2180-10-298-S2.DOC]

**Table 2.**

|  | *Sph*I | *Nco*I | *Nhe*I | *Ssp*I | *Sfu*I | *Eco*RV | *Dra*I | *Vsp*I | *Hinc*II | *Eco*RI | *Hind*III | *Avr*II |
| --- | --- | --- | --- | --- | --- | --- | --- | --- | --- | --- | --- | --- |
| ***Lactobacilii*** |  |  |  |  |  |  |  |  |  |  |  |  |
| *L. acidophilus* | *-* | *+* | *-* | *-* | *-* | *-* | *-* | *-* | *-* | *+* | *+* | *+* |
| *L. agilis* | *+* | *-* | *+* | *+* | *-* | *-* | *-* | *+* | *-* | *-* | *-* | *-* |
| *L. amylovorus* | *-* | *+* | *-* | *-* | *+* | *-* | *-* | *-* | *-* | *+* | *+* | *+* |
| *L. casei* | *-* | *-* | *-* | *-* | *-* | *+* | *+* | *+* | *-* | *-* | *+* | *-* |
| *L. ferintoshensis* | *+* | *-* | *-* | *+* | *-* | *-* | *-* | *+* | *+* | *-* | *+* | *-* |
| *L. fermentum* | *+* | *-* | *+/-* | *-* | *-* | *-* | *-* | *+* | *-* | *-* | *-* | *-* |
| *L. hilgardii* | *+* | *-* | *-* | *+* | *-* | *-* | *-* | *+* | *-* | *-* | *+* | *-* |
| *L. manihotivorans* | *+* | *-* | *+* | *-* | *+* | *+* | *-* | *-* | *-* | *-* | *+* | *-* |
| *L. mucosae* | *+* | *-* | *-* | *+* | *-* | *-* | *-* | *+* | *-* | *-* | *-* | *-* |
| *L. nagelli* | *+* | *-* | *-* | *-* | *-* | *-* | *-* | *-* | *-* | *-* | *-* | *-* |
| *L. paracasei* | *+* | *-* | *-* | *-* | *-* | *+* | *+* | *+* | *-* | *-* | *+* | *-* |
| *L. plantarum* | *+* | *-* | *-* | *+* | *+/-* | *-* | *-* | *+* | *+* | *-* | *-* | *-* |
| *L rhamnosus* | *-* | *-* | *-* | *-* | *-* | *+* | *+* | *-* | *-* | *-* | *+* | *-* |
| *L. vini* | *+* | *-* | *-* | *-* | *-* | *-* | *+/-* | *-* | *-* | *+* | *-* | *-* |
| *L. diolivorans like* | *-* | *-* | *-* | *+* | *-* | *-* | *-* | *+* | *-* | *-* | *+* | *-* |
| *Non-Lactobacilii* |  |  |  |  |  |  |  |  |  |  |  |  |
| *Oenococcus kitaharae-like* | *-* | *-* | *-* | *-* | *+* | *+* | *+* | *+* | *-* | *-* | *-* | *-* |
| *Weissella paramesenteroides* | *-* | *-* | *-* | *-* | *+* | *-* | *-* | *-* | *+* | *-* | *-* | *-* |
